# The Aryl Hydrocarbon Receptor as a Modulator of Anti-viral Immunity

**DOI:** 10.3389/fimmu.2021.624293

**Published:** 2021-03-05

**Authors:** Maria Florencia Torti, Federico Giovannoni, Francisco Javier Quintana, Cybele Carina García

**Affiliations:** ^1^Laboratory of Antiviral Strategies, Biochemistry Department, School of Sciences, University of Buenos Aires, IQUIBICEN-Consejo Nacional de Investigaciones Científicas y Técnicas, Buenos Aires, Argentina; ^2^Ann Romney Center for Neurologic Diseases, Brigham and Women's Hospital, Harvard Medical School, Boston, MA, United States

**Keywords:** aryl hydrocarbon receptor, viral infections, RNA viruses, DNA viruses, host response

## Abstract

The aryl hydrocarbon receptor (AHR) is a ligand-activated transcription factor, which interacts with a wide range of organic molecules of endogenous and exogenous origin, including environmental pollutants, tryptophan metabolites, and microbial metabolites. The activation of AHR by these agonists drives its translocation into the nucleus where it controls the expression of a large number of target genes that include the AHR repressor (AHRR), detoxifying monooxygenases (CYP1A1 and CYP1B1), and cytokines. Recent advances reveal that AHR signaling modulates aspects of the intrinsic, innate and adaptive immune response to diverse microorganisms. This review will focus on the increasing evidence supporting a role for AHR as a modulator of the host response to viral infection.

## Introduction

Viral infectious diseases are a major cause of death and disability for millions of people throughout the world. Many factors, from host gender, age, genetics, up to nutritional status play a role in determining the susceptibility to and pathophysiological consequences of infection. In the end, the clinical outcome is variable and greatly dependent on the net result of the damage caused both by the pathogen as well as by the immune response of the host in response to the pathogen. Importantly, environmental factors, such as chemical exposures, also contribute to differential clinical outcomes of infections at the individual and populations level.

The aryl hydrocarbon receptor (AHR) is a ligand-activated transcription factor that interacts with a diverse array of anthropogenic and natural agonists (1–5). Because of the ubiquitous distribution of AHR agonists, we are constantly exposed to a diverse spectrum of AHR ligands; AHR signaling participates in our adaptation to changing environments as defined by alterations in the diet, the microbiome and metabolism.

Recent reports have shown a role for AHR as a modulator of the intrinsic, innate and adaptive immune response to viral infections (6, 7), with both positive and negative effects on host resistance and survival based on the experimental system used. In this review, we will evaluate the role of AHR on the host response to viral infection and its potential as a candidate target for therapeutic intervention.

## AHR Signaling

**Figure 1:**
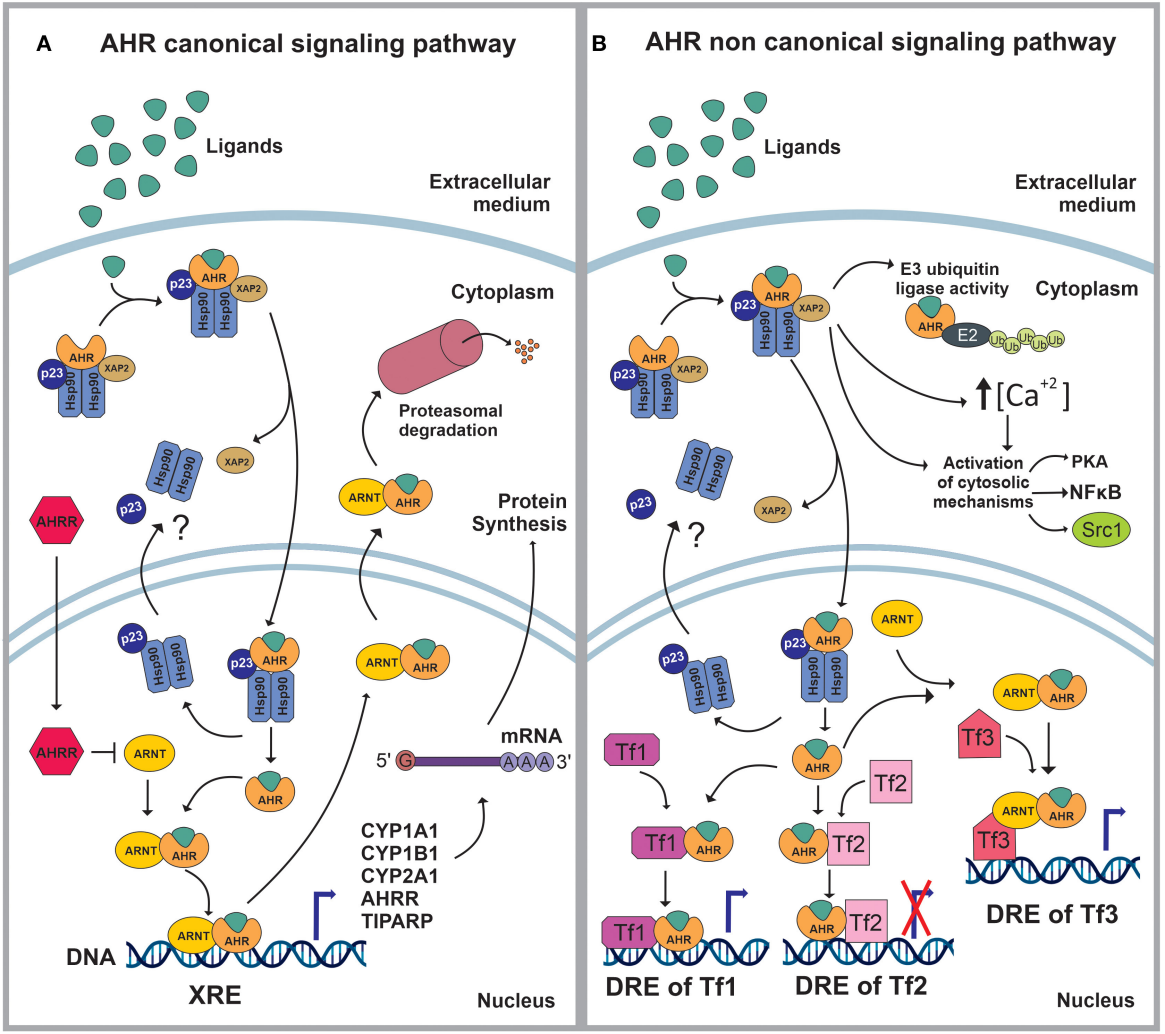
**(A)** In stationary state, the aryl hydrocarbon receptor (AHR) is part of a complex composed by the 90kDa heat shock protein (HSP90), the co-chaperone p23 (p23) and the hepatitis B virus X-associated protein (XAP2/AIP/ARA9). The complex stabilizes AHR conformation, protects AHR from proteolitic degradation and contributes to its subcellular localization. The ligand (L) binding triggers a conformational change in AHR exposing a nuclear localization signal (NLS). Then, XAP2 is released from the complex and the L-AHR-HSP90-p23 structure translocates into the nucleus via β-importins. Inside the nucleus, the chaperones are released from the complex and return to the cytoplasm whilst the AHR-L structure heterodimerizes with the aryl hydrocarbon receptor nuclear translocator (ARNT), interacts with its DNA- response-elements -xenobiotics response element (XRE)- and regulates the expression of different genes. The AHR canonical signaling pathway is characterized by the expression of CYP1A1, CYP1B1, CYP2A1, TIPARP and AHRR. Following the modulation of its target genes, the ARNT-AHR-L complex exits the nucleus and is targeted for proteasomal degradation. **(B)** The AHR non-canonical signaling pathway involves the regulation of cytoplasmic proteins as well as the control of gene expression. Within the cytoplasm, the AHR-L complex can function as an E3 ubiquitin ligase, promoting the proteasomal degradation of target proteins. It also increases the intracellular Ca2+ levels and interacts with different proteins such as PKA, NFκB and Src1. Once in the nucleus, AHR-L is capable of interacting and controlling the activity of other transcription factors (Tf) through transactivation/transrepression or it can exert a Co-activator/Co-Inhibitor role when it is associated with ARNT.

AHR is a member of the basic helix–loop–helix (bHLH)/PER-ARNT-SIM (PAS) superfamily of transcription factors (8). AHR is a well-conserved protein, with an ubiquitous presence in mammalian tissues, and variable expression levels among tissues and throughout life (9–11) AHR controls a broad range of biological processes in response to environmental and metabolic cues (9–11). When inactive, AHR is part of a stable cytoplasmic multiprotein complex composed by the chaperone heat-shock protein 90 (HSP-90), the co-chaperone p23 (p23) and the hepatitis B virus X-associated protein (XAP2) (12–21). This complex has been proposed to stabilize the conformation of AHR, protect it from proteolitic degradation and contribute to its subcellular localization. AHR canonical signaling pathway is triggered by the binding of an agonist (A), which triggers a conformational change that exposes AHR nuclear localization signal (NLS), resulting in the nuclear translocation of the A-AHR-HSP90-p23 complex via β-importins. There is still some discrepancy over the role of XAP2 on nuclear translocation. Some studies suggest that XAP2 is involved in the cytoplasmic anchorage of the AHR complex. However, other studies suggest that XAP2 interferes with the interaction of the NLS with β-importins; this last interpretation would require the XAP2 to be released from the AHR complex before the nucleocytoplasmic shuttling occurs (12–16, 21–28). Inside the nucleus, the chaperones disassemble from the complex, the AHR-A structure heterodimerizes with the AHR nuclear translocator (ARNT) and interacts with specific sequences in DNA (xenobiotics response element, XRE) to control the expression of target genes (12, 13, 19, 21, 29, 30). Among these target genes are those encoding the Cytochrome P450 enzymes, specifically, members of the families 1 and 2: CYP1A1, CYP1B1, and CYP2A1 (21, 30) (Figure 1A).

AHR also controls biological processes through a non-canonical signaling pathway, which involves multiple molecular mechanisms. For instance, AHR activation can result in the increase of the intracellular concentrations of Ca^+2^, or the activation of Src tyrosine kinase and focal adhesion kinase. Regarding the non-canonical genomic regulation, it has been reported that AHR can heterodimerize with other nuclear proteins, including transcription factors, to modulate their activity by transactivation/transrepression or protein-protein interactions. In this context, AHR has been shown to cross-talk with the nuclear factor kappa light chain enhancer of activated B cells (NFκB), activator protein-1 (AP-1), estrogen receptor (ER) and glucocorticoid receptor (GR), Krüppel-like Factor 4 and 6 (KLF4, KLF6), signal transducers and activators of transcription (STAT) proteins and members of the CCAAT-enhancer-binding proteins (C/EBP) family (2, 20, 21, 30–34) (Figure 1B).

**Figure 2:**
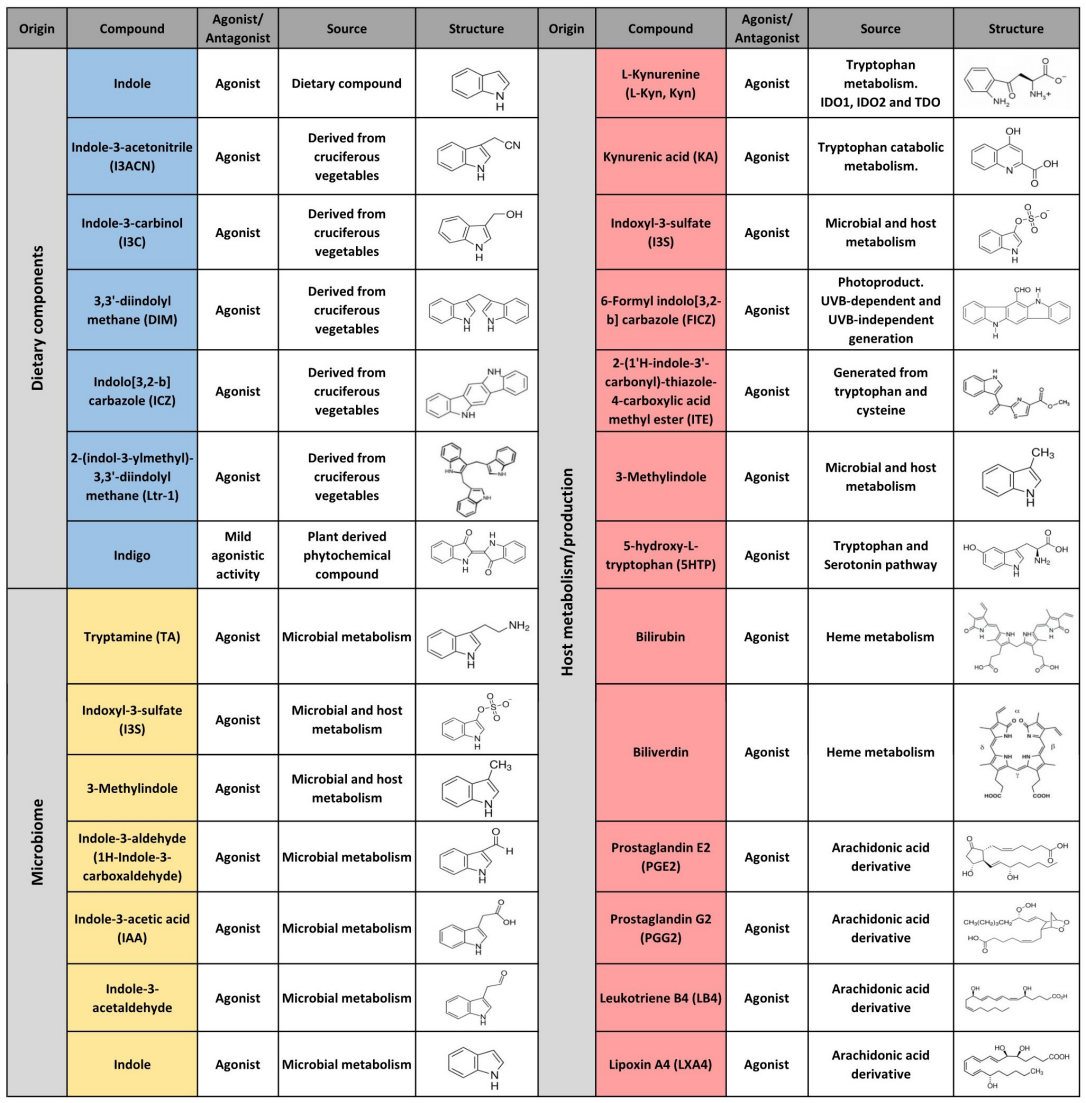
Selected AHR endogenous agonists.

**Figure 3:**
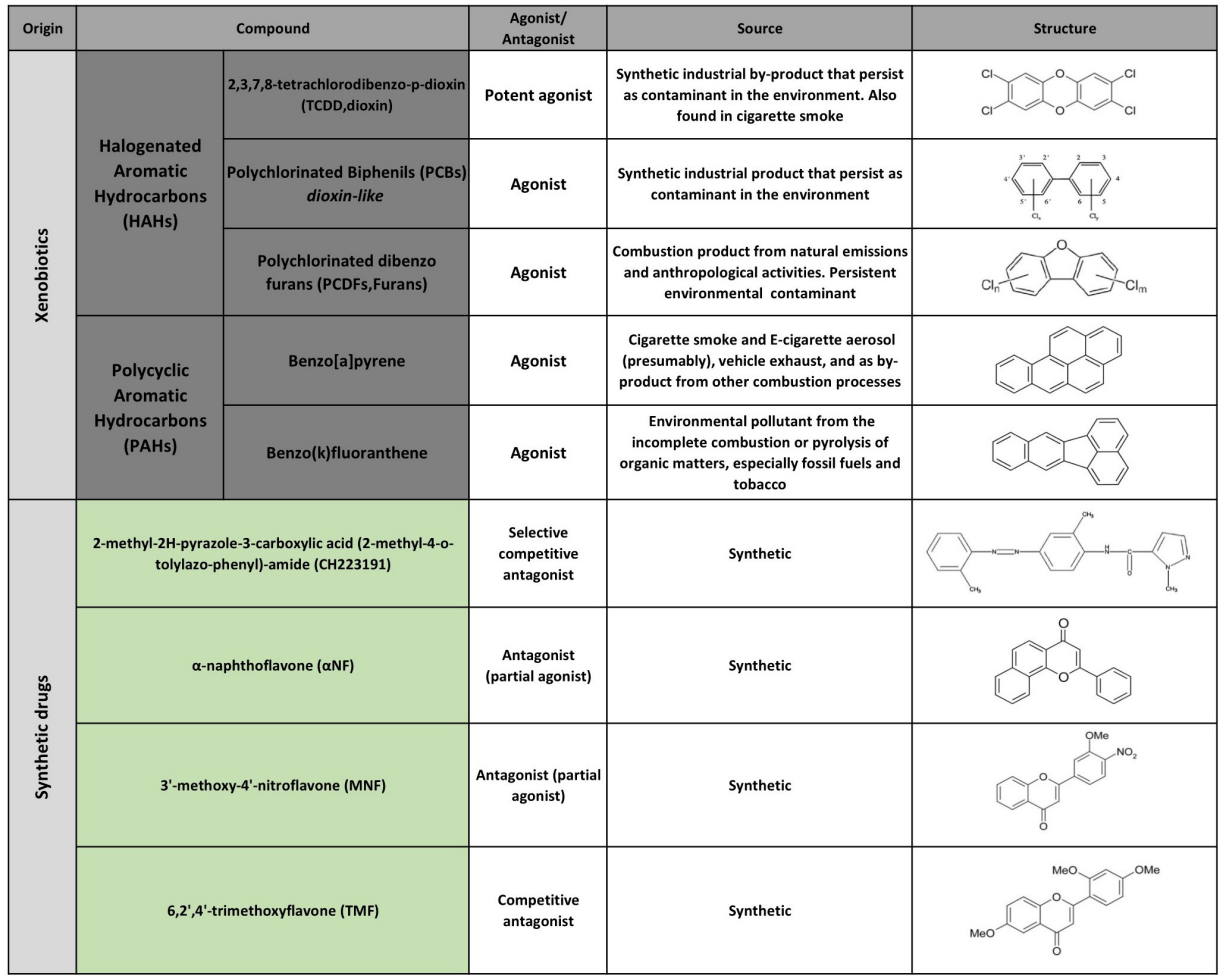
Selected AHR exogenous agonists and antagonists.

The first AHR agonists identified were the non-halogenated polycyclic aromatic hydrocarbons (PAH) and halogenated aromatic hydrocarbons (HAH), which are major anthropogenic pollutants. These compounds are quite abundant and persistent in the environment due to their long half-life and bioaccumulation in the trophic chain. Their toxicity has been largely documented in humans as well as in other species with estimates indicating that more than 90% of the human exposures occur via contaminated food (21, 35–41). However, over the last decades, a wide variety of agonists from multiple sources such as the environment, the microbiome, the diet and metabolism have been shown to activate AHR. Currently they are classified into natural or synthetic, endogenous or exogenous agonists depending on their nature and sources (Figures 2, 3) (2, 21, 42, 43).

Multiple AHR modulators are used to study the function of this pathway in the control of immunity and other biological processes. The most utilized AHR agonists include the 2,3,7,8-tetrachlorodibenzo-p-dioxin (TCDD), an industrial by-product that persists as contaminant in the environment and is well-known for its toxicity in humans and other species; the 6-formylindolo(3,2b)carbazole (FICZ), an endogenous tryptophan (Trp) photoproduct; the endogenous indole derivative indoxyl 3-sulfate (I3S) and kynurenine (Kyn), a metabolite from the Trp catabolic pathway product from the enzymatic activity of the tryptophan 2,3-dioxygenase-2 (TDO2) and indoleamine 2,3-dioxygenase−1/2 (IDO-1/IDO-2) (Figures 2, 3). IDO-1 is expressed in response to IFN-γ stimulation and is thought to contribute to IFN-γ antiviral activity by the depletion of L-Trp during Kyn generation (44–47). Kyn produced by IDO-1 can then activate AHR, which can further boost IDO-1 expression, establishing an IDO1-AHR-IDO1 positive feedback loop to prolong AHR activation (21, 48).

Conversely 2-methyl-2H-pyrazole-3-carboxylic acid (2-methyl-4-o-tolylazo-phenyl)-amide (CH223191) is a synthetic potent and specific AHR competitive antagonist that preferentially inhibits the response to TCDD and related HAHs (Figure 3).

## Environmental Factors and AHR

AHR agonists are incorporated from the environment via the oral route (e.g., dietary agonists), and also via their inhalation and absorption across mucosal barriers (1, 5, 49, 50). The environmental levels of AHR-activating pollutants (i.e., dioxins, PAH and HAH) are being reduced in the most developed countries, but their levels are increasing in the developing world (51–54). Therefore, these AHR agonists remain a continued threat to public health. Moreover, epidemiologic and animal-model studies suggest that environmental chemicals influence host responses to infectious diseases (55), and strong associations have been described between dioxin and HAH levels and viral respiratory tract infections, wheezing, and poor vaccine responses in infants and children (56–62). Hence, based on its multiple effects on the immune response, the modulation of AHR signaling by environmental chemicals is likely to have important effects on the host response to viral infection.

## AHR Variants in Human Diseases

AHR regulates numerous genes associated with cellular homeostasis, proliferation, immune maturation and function, hematopoietic stem cell expansion, glucose tolerance, and the development of many human diseases. Indeed, accumulating evidence supporting a role for AHR signaling in various diseases has encouraged the investigation of the potential impact of AHR genetic variants in the susceptibility and development of human disorders. For instance, a splicing variant of AHR has been associated with retinitis pigmentosa (63). In addition, single-nucleotide polymorphisms (SNPs) in *AHR* were identified as risk factors for the development of lung cancer in a population of Chinese cigarette smokers (64); other AHR SNPs were recently associated with higher risk of Crohn's disease (65, 66). In addition, AHR genetic variants have been linked to endocrine disorders such as cyclical Cushing's disease (67) and to acromegaly or somatotropinoma (68, 69). Finally, *AHR* variants have been identified as risk factors for the development of coronary arterial (70) and heart disease (71). Taken together, these studies highlight the potential contributions of AHR polymorphisms to human disease, and call for future studies focused on the investigation of the role of these polymorphisms in viral infections.

## AHR and Viral Infections

### RNA Virus Infections

#### Influenza Virus

Evidence showing that AHR activation has a strong influence on host resistance to viral infection was first reported over 40 years ago (72, 73). Those studies concluded that even very low doses of the AHR agonist TCDD enhance morbidity and mortality in rats and mice infected with lethal strains of influenza A virus (IAV) (74, 75). Influenza viruses are negative-sense single- stranded RNA viruses belonging to the *Orthomyxoviridae* family. IAVs cause acute respiratory infections in humans and are a great burden to public health and the global economy. As a result of the 2009 H1N1 pandemic, more than 120,000 people died worldwide, the majority of which were in the young age range (<65 years old) (reported by CDC). Avian influenza strains such as the H5N1 and H7N9 have also raised concern for future pandemics due to their capacity to cross the species barrier and cause lethal infections in humans. Although vaccination against seasonal influenza is an essential part of the public health strategy, its efficacy is variable, and there are only few therapeutic options for people who become infected.

Host defense during a primary IAV infection is mainly promoted by virus-specific CD8^+^ cytotoxic T lymphocytes (CTL), which kill infected cells in the lung (76). At this stage, B cells do not play a major role as they do not produce virus-specific antibodies. However, class-switched B cells, antibody-secreting plasma cells, and memory B cells participate in the generation of antibodies that protect against repeated infection with homotypic virus strains (77). It has been reported that AHR is highly induced upon B cell activation and has a critical role in regulating activation-induced cell fate outcomes. Of note, AHR suppresses antibody class switching *in vivo* after IAV infection and immunization with model antigens (78).

In addition, IAV-specific CD4^+^ T cells are key for the generation of virus-specific antibodies by B cells and the establishment of immunologic memory; additional functions are played by Foxp3^+^CD25^+^ regulatory CD4^+^ T cells (Tregs), Th17, and T follicular helper (T_FH_) cells during virus infection (79–82). Importantly, early life exposure to chemical AHR ligands alters the CD4+ T cell responses to IAV infection in adulthood (83). Finally, upon successful viral clearance, a pool of memory lymphocytes remains, which secures a rapid response if the same or similar influenza virus strains are later encountered. The proper regulation of these cell populations is required to generate an immune response which can successfully resolve the viral infection while averting an excessive inflammatory response with its associated immunopathology.

Treatment with a single oral dose of TCDD has been shown to increase morbidity and mortality in mice infected with a sublethal inoculum of IAV. The adaptive immune response is impaired upon AHR activation, with a marked reduction in dendritic cell (DC) function, the expansion and differentiation of CD4^+^ and CD8^+^ T cells, and virus-specific IgG titers (84). Recent genome wide transcriptional analyses of DCs isolated from lungs of IAV-infected mice treated with TCDD detected a strong down-regulation of CD209a and CCL17 expression. Indeed, Ingenuity Pathway Analysis (IPA) revealed that the most altered signaling pathways in these DCs are related to immune cell trafficking, cellular movement and hematological system development and function (85). Interestingly, although AHR activation increases morbidity and mortality caused by IAV infection, the kinetics of viral growth and the efficacy of the viral clearance, are not significantly different between control and TCDD-treated mice in primary infections nor even during homotypic reinfections (86–89). These findings suggest that the dysregulated expansion of the virus itself is not to blame for the poorer outcomes of TCDD-treated mice. Indeed, AHR activation increases the recruitment of neutrophils to the infected lung, and elevates the expression of IFNγ and the inducible nitric oxide synthase (iNOS) in the lungs during IAV infection (90, 91). These molecules could participate in antiviral mechanisms, but their dysregulated activity promote immunopathology which results in poorer survival. Indeed, neutrophil depletion markedly improves survival and abrogates the enhancement of broncho-pulmonary inflammation triggered by AHR activation with TCDD during acute IAV infection (92).

Studies using AHR mutant mice suggest that changes in the host response to IAV are mainly driven by direct interactions of AHR with XREs (85, 91). However, the specific AHR gene targets involved in the altered host responses to IAV induced by TCDD remain to be determined. Recent reports based on adoptive transfer, bone marrow transplantation, and conditional gene ablation have shown that AHR modulates both the response of hematopoietic cells, endothelial cells, and lung epithelial cells to IAV (93), suggesting that CD8^+^ T cell responses to IAV are suppressed by AHR signaling via indirect mechanisms (94). In addition, the increase in neutrophils and iNOS and IFNγ expression in the lungs of TCDD-treated mice is independent of AHR expression in hematopoietic cells (93). Instead, arh knockout mice revealed that the increased pulmonary neutrophilia induced by TCDD requires AHR activation in the respiratory epithelium, while the increase in iNOS expression is dependent on AHR activation in endothelial cells (91). Hence, multiple cell types participate in the AHR-mediated alterations of the host response to IAV.

Further insights were provided by the comparison of the alterations induced in the immune response to IAV by four representative AHR agonists: (1) TCDD, (2) 3,3′,4,4′,5-pentachlorobiphenyl (PCB126), a pollutant with documented human exposure, (3) 2-(1′H-indole-3′-carbonyl)-thiazole-4-carboxilic acid methyl ester (ITE), an AHR agonist isolated from mucosal tissues, and (4) FICZ, a degradation product of Trp. All these AHR ligands diminished virus-specific IgM levels and increased the proportion of regulatory T cells (7). TCDD, PCB126 and ITE, but not FICZ, reduced virus-specific IgG levels and CD8^+^ T cell responses. Similarly, ITE, PCB126, and TCDD reduced Th1 and T follicular helper cells, whereas FICZ increased their frequency. In *Cyp1a1*-deficient mice, all compounds reduced the response to IAV. *Ahr* knockout mice denoted that these compounds require AHR within hematopoietic cells to exert their effects (7). These findings suggest that the differential effects of specific AHR agonists on the immune response to IAV reflect differences in the half-life of the agonists, and potentially the induction of different AHR conformations. A deeper understanding of the mechanisms behind these ligand-specific effects will pave the way for the design of AHR-targeted therapeutics.

#### Coronaviruses

Coronaviruses (CoVs) are a family of positive-sense single-stranded RNA viruses with public health and agricultural importance. They mostly cause enteric or respiratory disease, which can be severe and life threatening. Human CoVs first come under the spotlight when outbreaks of the severe acute respiratory syndrome (SARS-CoV-1) and the Middle East respiratory disease (MERS-CoV) were reported in 2002-03 and 2012, respectively. At the end of 2019, a new human CoV (SARS-CoV-2) was identified in Wuhan, China, and associated with a severe respiratory infection, known today as CoVs disease 2019 (COVID-19). Compared to other positive-sense RNA viruses, CoVs have an exceptionally large genome (30 kb) and employ a complex genome expression strategy (95); our knowledge of host factors involved in CoVs replication is still extremely limited.

It was recently reported that AHR is activated in cells infected with a prototypic CoV, mouse hepatitis virus (MHV), resulting in the expression of several effector genes (96). Indeed, AHR was shown to be important for modulation of the host immune response to MHV, playing a role in the expression of the downstream effector TCDD-inducible poly(ADP-ribose) polymerase (TiPARP), which is required for maximal viral replication. In accordance with this, knockdown of TiPARP reduced viral replication and increased IFN expression, suggesting that TiPARP is a proviral factor for MHV infection. Moreover, MHV replication induced the expression of other AHR-driven genes in macrophages and DCs of infected mice. The pharmacologic modulation of AHR activity regulated the expression levels of cytokines induced by infection, specifically, interleukin 1β (IL-1β), IL-10, and TNF-α, supporting a role for AHR activation in the host response to MHV infection. Of note, while IDO-1 drives AHR activation in the context of other infections, MHV induced a similar expression level of downstream genes in wild-type and IDO-1-/- macrophages, suggesting that additional pathways besides IDO-1 are involved in AHR activation.

In the context of the explosive amount of research performed on SARS-CoV-2, a model was proposed where SARS-CoV-2 would activate AHR by an IDO1-independent mechanism, initially bypassing the IDO1-Kyn-AHR pathway, and then AHR would enhance its own activity through an IDO1-AHR-IDO1 positive feedback loop prolonging activation induced by this novel pathogen (97). In this sense, researchers discussed the possibility of a direct activation of AHRs by CoVs inducing immediate and simultaneous up-regulation of diverse AHR-dependent downstream effectors, which in turn, would result in AHR-related syndromes, consisting of inflammation, thromboembolism, and fibrosis, finally culminating in multiple organ injuries, and death (98).

The activation of AHRs by CoVs may lead to a diverse set of phenotypic disease scenarios depending on the time after infection, overall state of health, hormonal balance, age, gender, co-morbidities, diet and environmental factors modulating AHR signaling. In addition, infection by SARS-CoV-2 or the non-related respiratory syncytial virus (RSV), results in increased AHR and IDO-1 lung expression, concomitant with increased pro-inflammatory gene expression, activation of the Tissue Factor/Plasminogen Activator Inhibitor-1 (TF/PAI-1) signaling pathway, and up-regulation of CYP1A1 (98). Finally, bioinformatic screens of novel approaches for the therapeutic modulation of AHR signaling established that dexamethasone may down-regulate both AHR and IDO-1 expression, while calcitriol/vitamin D_3_ may down-regulate AHR, and tocopherol/vitamin E may down-regulate IDO-1 (98).

#### Flavivirus Infections

Flaviviruses comprise a group of positive-sense single-stranded RNA viruses of ~9–13 kb that cause severe endemic infection and epidemics on a global scale. Representative members of this group include dengue, West Nile, and Zika viruses. Flaviviruses constitute a significant health issue worldwide and many members of this family have shown potential to emerge and cause outbreaks in non-endemic geographical regions. Additionally, reemergence in areas where circulation was previously thought to be contained has been observed, such as the case of the 2018 outbreak of yellow fever virus in Brazil. Other medically-important flaviviruses such as Japanese encephalitis virus, which circulates mainly in Southern and Southeastern Asia, or tick borne encephalitis virus, which is endemic in parts of Eurasia, have not yet expanded globally. However, because their vectors are widely distributed, they do have potential for spreading. Of note, other worldwide human important diseases associated with this family include hepatitis C virus (99).

##### Zika Virus

Zika virus (ZIKV) is a mosquito-vectored flavivirus isolated in 1947 in Uganda. Its circulation has been reported in humans from West Africa and Asia since the mid-1950s (100). ZIKV infection used to be a neglected disease for most of its history due to the mildness of its symptoms and the fact that it was geographically restricted. However, in 2015 Brazil registered an unprecedented epidemic (101) characterized by a high incidence of microcephaly cases, formally accepted to be linked to ZIKV in April 2016. The mechanism by which ZIKV crosses the placenta is still unclear, but its neurotropism and ability to destroy neural cells have been well-established (102). Neural progenitor cells (NPCs) are the primary target of the ZIKV, and this may partly explain the high number of abnormalities detected in neuroimaging examinations (103). It was reported that environmental factors strongly correlate with nutrition and socioeconomic position affecting the immune status and response to ZIKV infections (85). When analyzing this relationship for Recife (Pernambuco, Brazil), a city that was severely hit by ZIKV, it was found that cases of reported microcephaly in 2015 and 2016 were largely concentrated in areas with more impoverished living conditions (104). Noticeably, AHR is highly expressed in the human placenta and its expression is upregulated in placentas of women suffering unexplained miscarriages (105, 106). Hence, it is conceivable that sustained AHR activation in women exposed to pollutants and living in impoverished conditions with degraded housing and malnutrition (107, 108) might increase their susceptibility to ZIKV infection and ZIKV congenital syndrome.

Consistent with these observations, we recently showed that ZIKV infection up-regulates IDO-1 and AHR expression in first trimester trophoblast cells (109) and NPCs (106). Indeed, AHR was identified as a key proviral factor for ZIKV infection. In addition, it was found that ZIKV infection triggers AHR activation, limiting the production of IFN-I, involved in antiviral immunity and favoring viral replication *in vitro*. Importantly, the relevance of these findings was further evaluated using an *in vivo* murine model, in which AHR pharmacologic inhibition blocked ZIKV replication and ameliorated newborn microcephaly. These results suggest that AHR is a candidate target for host-directed therapies for flavivirus infection.

##### Dengue Virus

Dengue virus (DENV) is a *flavivirus* endemic in many tropical and sub-tropical countries where the transmission vectors *Aedes* spp. mosquitoes are present. There are four serotypes of DENV (DENV1-DENV4). Each serotype is antigenically different, meaning they elicit heterologous antibody responses. Infection with one serotype elicits neutralizing antibodies to that serotype. Cross-protection from infection with other serotypes is short lived; instead heterotypic infection can cause severe disease. After DENV infection, activation of innate immune pathways occurs, including IFN-I, complement, apoptosis, and autophagy, which the virus can evade or exploit to exacerbate disease.

With regards to the impact of AHR modulation on DENV infection, only *in vitro* data is available, suggesting that the pharmacological inhibition of AHR suppresses DENV replication (106). AHR activation in A549 cells with I3S increased DENV2 yield, as determined by standard plaque assay. Conversely, AHR inhibition with the antagonist CH223191 reduced viral RNA level, viral protein expression and viral titer in culture supernatants. Moreover, the knockdown of AHR diminished the production of DENV2 infectious viral particles. Of note, the treatment with the AHR antagonist CH223191 prior the infection with DENV1, DENV3, and DENV4, showed a comparable antiviral effect to the one already described for the best characterized serotype, DENV2 (106). The four dengue serotypes usually co-exist in the same geographical regions, which also leads to increased disease severity mediated by antibody-dependent enhancement (110). Also, different flaviviruses (e.g., ZIKV, yellow fever) may co-exist in the same regions as well. Therefore, the development of a single drug effective against many flaviviruses represents an example of a “one-drug, multiple bugs” approach (111) which may translate into major benefits, including: (i) the ability to treat future yet-unknown flavivirus outbreaks, (ii) the potential administration of a drug even before a differential diagnosis between flaviviruses can be made, and (iii) much lower research and development costs.

##### Hepatitis C Virus

Hepatitis C virus (HCV) is a flavivirus belonging to the *Hepacivirus* genus. HCV infection can result in a persistent disease (112), remaining asymptomatic for years before the development of severe liver pathology including cirrhosis and hepatocellular carcinoma. HCV regulates critical signaling pathways in hepatocytes, and actively evades the antiviral immune response. In particular, HCV modulates cell metabolism and remodels specialized membrane structures and organelles such as double-membrane vesicles and lipid droplets, favoring virus replication and virion assembly. However, the molecular bases of these host-virus interactions are still unclear. Recently it was demonstrated that the benzamide derivative flutamide, which has been shown to act as an AHR antagonist (113), inhibits the cellular capacity to produce infectious HCV particles (113). Flutamide blocks the biogenesis of lipid structures in HCV-infected cells, disrupting virion assembly, indicating that AHR plays a key role in modulating LD storage and HCV infection. This novel role of AHR in lipid biogenesis was also confirmed in non-infected Huh-7 cells and primary human hepatocytes, suggesting that AHR regulates store lipid reserves independently of viral infection. Indeed, the product of the prototypic AHR target gene *CYP1A1* was identified as the main regulator of AHR-mediated lipid biogenesis (113). Indeed, the inhibition of AHR-induced *CYP1A1* up-regulation diminished the lipid droplet enlargement. Conversely, the enhanced expression of CYP1A1 restored lipid reserves in AHR-inhibited cells. Altogether, these data identify a role for AHR in the control of lipid biogenesis, a hallmark of HCV infection that boost the production of viral particles, identifying AHR as a candidate therapeutic target for HCV infection.

#### Retroviruses

Human immunodeficiency viruses (HIV) belong to *Retroviridae* family, *Orthoretroviridae* subfamily in *Lentivirus* genus. They harbor spherical particles surrounded by an envelope whose outer part contains glycoproteins. The genome consists of two positive-sense single-stranded RNA copies and can be transcribed into double-stranded DNA by a reverse transcriptase, an enzyme contained in the viral particle. This provirus can integrate into the host cell chromosome until the initiation of a replication cycle (114).

The acquired immunodeficiency syndrome (AIDS) caused by HIV-1 is a progressive condition in which virus-induced immune dysfunction results in the development of serious opportunistic infections and cancers. Several metabolic pathways are altered by HIV-1 infection, with an impact on immune activation, inflammation, and acquisition of non-AIDS co-morbid diseases. Unusual high levels of Kyn have been detected in association with accelerated HIV-1 pathogenesis, but the molecular mechanism behind this observation remains unclear. However, it was recently reported that AHR is activated by Trp metabolites to favor HIV-1 infection and reactivation (115), suggesting a novel role for AHR in AIDS. AHR directly binds to the HIV-1 5′ long terminal repeat (5′-LTR) to activate viral transcription. Moreover, the binding of AHR with Tat viral protein facilitates the recruitment of positive transcription factors to viral promoters. These findings elucidate a previously unappreciated mechanism through which cellular Trp metabolites affect HIV pathogenesis, and also suggest that AHR signaling may be targeted to modulate HIV-1 infection (115).

In another set of studies, *in vitro* treatment with TCDD, benzo[a]pyrene, and 3-methycholanthrene increased HIV gene expression and the level of secreted p24 viral protein in several different cell lines; the use of mutant and dominant negative AHR constructs suggest that these effects on HIV-1 are AHR-dependent. Interestingly, the simian retrovirus SIV Vpx protein provides complete and partial resistance to the antiviral effects of AHR (116). Interactions between AHR and NF-κB have been implicated in some, but not all, of these studies (117–120), leaving the molecular mechanisms by which AHR impacts viral latency and viral replication, as well as their *in vivo* relevance, uncertain.

### DNA Virus Infections

#### Herpesviruses

The large family of DNA genome *Herpesviridae* causes infections and diseases in multiple animal species, including humans. In particular, five herpesviruses are extremely widespread among humans: HSV-1 and HSV-2 (causing orolabial and genital herpes), varicella zoster virus or HHV-3 (causing chickenpox and shingles), Epstein–Barr virus or HHV-4 (responsible for several diseases, including mononucleosis and some cancers), and cytomegalovirus or HHV-5. More than 90% of adults have been infected with at least one of these pathogens (121–126). In addition, Kaposi's sarcoma-associated herpesvirus (KSHV), also known as HHV-8 is an important human health problem among immunocompromised people.

##### Herpes Simplex Virus-1

Ocular HSV-1 infection can result in chronic cornea inflammation driven by conventional CD4^+^ T cells and neutrophils, which ultimately leads to blindness. A recent study showed that TCDD reduces effector Th1 and Th17 cells, neutrophilic inflammation, and increases Foxp3^+^ Tregs in a mouse model of ocular HSV-1 infection (127). In this HSV-1 model, TCDD-treated mice harbored higher virus titers, and many succumbed to herpes encephalitis if AHR was activated before to infection. However, when AHR activation was triggered post HSV-1 infection, herpes encephalitis was reduced and there was improved pathology in the eye tissue. Hence, the timing of AHR activation seems to control the balance between limiting immunopathology and eliminating anti-virus protective immunity.

##### Human Cytomegalovirus

Human cytomegalovirus (HCMV), a beta-herpesvirus, causes severe birth defects in newborn infants and serious disease in immunocompromised patients (128). To replicate, HCMV must interfere with cellular DNA replication and block the cellular innate immune response, specifically IFN production and the expression of interferon-stimulated genes (ISGs). IDO-1 activation is believed to restrain HCMV multiplication by depriving the infected cells of Trp, while it can also limit virus-induced immunopathology via the production of Kyn. Indeed, elevated Kyn levels are detected during HCMV replication, high plasma Kyn levels have been correlated with HCMV reactivation in renal transplant recipients, and AHR activation has been shown to promote viral replication (129). Moreover, recent reports have described the interplay between AHR signaling and HCMV replication: (i) HCMV infection of primary human fibroblasts triggers the persistent induction of AHR transcriptional activity; (ii) Sustained AHR activity is associated with tightly balanced IDO-1 activity; (iii) AHR signaling is required for the efficient replication of virus; (iv) HCMV induced G1/S cell cycle arrest depends on AHR activity; and (v) HCMV exploits AHR signaling to counteract the innate antiviral immune response, in a negative feedback loop containing IDO1-Kyn-AHR (129). Follow up studies are needed to establish the molecular mechanisms by which endogenous AHR signaling modulates HCMV infection.

##### Epstein Barr Virus

A relationship between Epstein Barr virus (EBV) and exposure to environmental AHR agonists has been proposed as a risk factor for non-Hodgkin lymphoma and other diseases (130, 131). Independent studies also detected a link between AHR and EBV genes and/or proteins. For example, the nuclear protein EBNA-3, which contributes to the transformation of EBV-infected B cells (132) interacts with AHR and the AHR chaperone protein XAP-2 (133, 134). However, the mechanistic roles of these associations in diseases such as non-Hodgkin lymphoma remains to be revealed. Surprisingly, it has been reported that EBNA-3 interacts with AHR in a ligand-independent manner. However, EBNA-3 boosts the TCDD-induced transcription of an AHR-driven reporter gene, suggesting that AHR activation synergizes with the effects of EBNA-3 during the control of AHR-target genes. The molecular mechanisms underlying the functional interactions between EBNA-3 and AHR might involve XAP2, which retains AHR in the cytoplasm in the absence of an exogenous ligand. Additional mechanisms might be involved, because XAP2 translocates to the nucleus in the presence of EBNA-3, which suggests that EBNA-3 might stabilize transcriptionally active AHR in the nucleus. Of note, these reports are the first to describe the physical interaction of AHR with a viral protein, identifying new roles and molecular mechanisms for AHR in virus-host interactions.

## Therapeutic Modulation of AHR Activity in Clinical Practice

As discussed in the previous sections, the available data support a role for AHR in the regulation of the host immune response against many viruses. Because AHR activity can be regulated by small molecules, AHR is an attractive target for therapeutic immunomodulation in the context of virus infection. Indeed, in pre-clinical models, AHR antagonists were shown to ameliorate ZIKV congenital syndrome in mice (106), increased survival in IAV infected mice (135) and reduce lung pathology induced by SARS-CoV-2 in hACE2-transgenic mice (136). However, clinical studies evaluating the safety and efficacy of targeting AHR for the treatment of viral infections are lacking. Conversely, many studies have evaluated the AHR pathway as a candidate target for the treatment of other diseases such as cancer (137). Epacadostat (Incyte Corp), the first IDO1-selective inhibitor to be tested on large phase 3 trials, failed to demonstrate anticancer efficacy, but a second generation of IDO inhibitors are currently being investigated. Promising pre-clinical results have been obtained for many AHR antagonists, including HP163 (Hercules Pharmaceuticals, also successfully tested against ZIKV *in vivo*) and PX-A24590 (Phenex Pharmaceuticals). Moreover, two additional AHR antagonists are currently under clinical trials: BAY2416964 (Bayer) and IK-175 (Ikena Oncology). All the lessons being learnt from the development of drugs targeting AHR in the context of other diseases will likely impact the development of molecules targeting AHR to treat viral infections.

Antiviral drugs can be classified in two major categories: direct-acting antiviral agents (DAAs) or host-targeted antivirals (HTAs). While DAAs target virus components, HTAs target host molecules that impact viral replication. HTAs offer two major advantages over DAAs: i) They can target a broad range of viruses that require a specific host factor, and ii) They minimize the selection of drug-resistant virus strains. However, the major caveat of HTAs is the greater risk of cellular toxicity. In this context, the targeting of AHR is no different. In order to address the potential issue of cellular toxicity, the use of nanoparticles as a vehicle to deliver AHR modulators to specific cell types has been proposed (138, 139). This approach has been shown to minimize toxicity and maximize the therapeutic effect in the target cell types.

In summary, the AHR pathway has stepped into the spotlight for the treatment of several non-viral diseases. The growing amount of evidence on the role of AHR in virus-host interactions supports the development of AHR antagonists as a new family of HTAs.

## Conclusions

Human migration, urbanization, people agglomeration, environmental factors and reassortment across species are considered strong driving forces for viral re-emergence (140). One of the more astonishing aspects of the recent SARS-CoV-2 pandemic is the high level of variability among patients in terms of disease severity: while some patients remain asymptomatic, others require intensive care. Environmental factors interact with genetic factors to control the response to multiple challenges including viral infections. The collective of all exposures throughout an individual's lifetime could represent a possible underestimated factor which may contribute to the variations in disease severity. This “exposome” includes environmental toxins, pharmacological treatments, lifestyle choices, diet, etc. Technological advances and interdisciplinary experimental systems combined with rational bioinformatic approaches (141) have allowed us to initiate a comprehensive assessment of the exposome and the pathways involved in sensing it, but our knowledge in this area is still scattered.

The well-defined AHR pathway provides an excellent model to investigate the effects of the exposome on multiple aspects of physiology such as the response to viral infections. In addition, a deep understanding of the complex interactions between the viral pathogen and the immunological host response is required in order to develop antiviral treatments.

**Figure 4:**
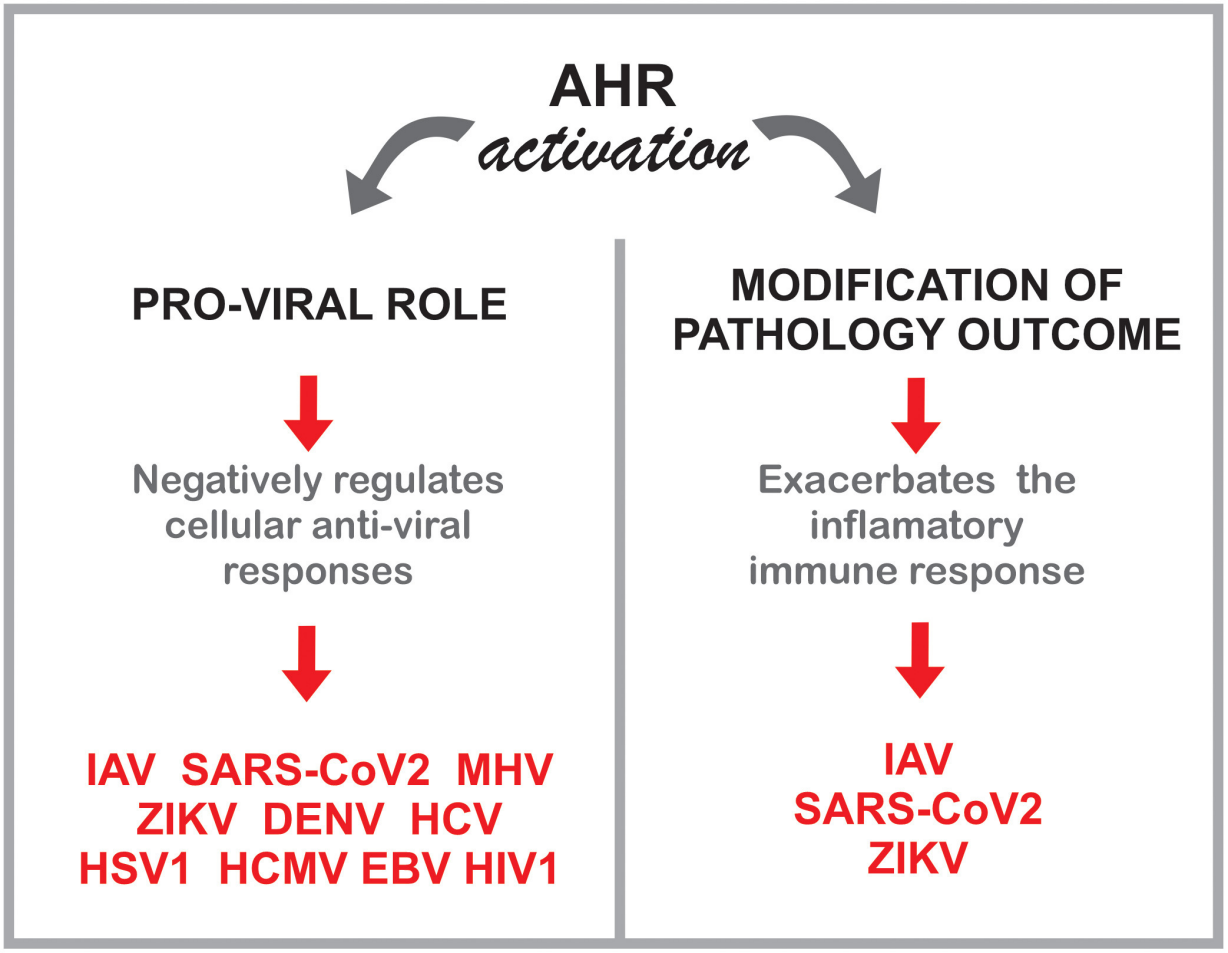
Potential roles of AHR during viral infections.

In this context, multiple studies indicate a role for the AHR environmental sensing molecule in the control of the adaptive, innate and intrinsic response to multiple common human viruses, including both RNA and DNA viruses (Figure 4). These findings may shed new light on the effects of the exposome on viral infections, while identifying novel approaches for therapeutic intervention.

## Author Contributions

MT, FG, CG, and FQ contributed conception, design, and wrote sections of the manuscript. All authors contributed to manuscript revision, read and approved the submitted version.

## Conflict of Interest

The authors declare that the research was conducted in the absence of any commercial or financial relationships that could be construed as a potential conflict of interest.
